# CRC-EBD: Epigenetic Biomarker Database for Colorectal Cancer

**DOI:** 10.3389/fgene.2020.00907

**Published:** 2020-10-06

**Authors:** Xingyun Liu, Xueli Zhang, Jing Chen, Benchen Ye, Shumin Ren, Yuxin Lin, Xiao-Feng Sun, Hong Zhang, Bairong Shen

**Affiliations:** ^1^Institutes for Systems Genetics, West China Hospital, Sichuan University, Chengdu, China; ^2^Center for Systems Biology, University, Suzhou, China; ^3^School of Medicine, Institute of Medical Sciences, Örebro University, Örebro, Sweden; ^4^Department of Ophthalmology, Guangdong Academy of Medical Sciences, Guangdong Provincial People's Hospital, Guangzhou, China; ^5^School of Science, Kangda College of Nanjing Medical University, Lianyungang, China; ^6^Department of Oncology and Clinical and Experimental Medicine, Linköping University, Linköping, Sweden

**Keywords:** colorectal cancer, database, epigenetics, DNA methylation, histone modification

## Introduction

Colorectal cancer (CRC) is one of the most common forms of cancer and a major cause of cancer-related death in both men and women worldwide (Lao and Grady, 2011; Siegel et al., 2017). Over 881,000 people globally have died from CRC, while 1.8 million were newly diagnosed with CRC in 2018 (Bray et al., 2018). The death rate of CRC has been steadily declining since 1990 (Siegel et al., 2017), but precise diagnosing and treating CRC remains challenging. Many patients exhibit few symptoms until the tumor has metastasized, making biomarkers for early diagnosis essential. Liquid biopsy is an easy and non-invasive method to detect ctDNA (circulating tumor DNA) in plasma or serum samples for early diagnosis, prognosis, or treatment (Tarazona and Cervantes, 2018). But ctDNA from a liquid biopsy is difficult to process and the lack of accuracy is still a problem (Kolencik et al., 2020); as a result, most previous studies focused on tumor tissue samples. For CRC patients, ctDNA is used to detect not only RAS mutations, but also DNA methylation, such as SEPTIN9 methylation (Song et al., 2018).

Epigenetic modifications play an important role in CRC genesis and progression (Danese and Montagnana, 2017). Epigenetics investigates heritable phenotype changes without alterations in the DNA sequence (Dupont et al., 2009). Epigenetic modifications include DNA methylation, histone modification, and genomic imprinting. DNA methylation is one of the best-characterized epigenetic mechanisms (Li and Zhang, 2014), which adds methyl groups to DNA, often at CpG sequences (Ehrlich et al., 1982). Emerging evidence suggests that some epigenetic modifications, DNA methylation in particular, can be important biomarkers for CRC (Ahmed, 2007). Aberrant DNA methylation is tissue-specific and often appears at early stages of cancer development (Jahn et al., 2011), making it a potentially ideal biomarker for early diagnosis of CRC.

We have previously constructed a biomarker database for colorectal cancer (CBD) (Zhang et al., 2018). Despite numerous reports on this subject so far, to our best knowledge, no database for cancer epigenetic biomarkers has been built yet. To enable the systematic study of epigenetics in CRC, we hereby established the first cancer epigenetic biomarker database, which was named CRC-EBD (Epigenetic Biomarker Database for Colorectal Cancer). CRC-EBD stores the epigenetic biomarkers information on CRC from PubMed literature. As precision medicine is becoming the new scientific paradigm (Morere, 2012), our database is built with more focus on collecting information regarding clinical samples in order to promote future translational researches on CRC.

## Materials and Methods

Data in CRC-EBD was manually collected from PubMed. We used “(colon[ti] OR rectosigmoid junction[ti] OR rectal[ti] OR anus[ti] OR bowel[ti] OR colorectum[ti] OR colorectal[ti]) AND (biomarker^*^[tiab] OR marker^*^[tiab] OR indicator^*^[tiab] OR predicator^*^[tiab] OR (drug target^*^[tiab]) OR (therapeutic target^*^[tiab])” as the term to search the PubMed for the CRC biomarkers. In addition, we used the keyword “AND methylat^*^[tiab]” for methylation biomarker, “AND histone^*^[tiab]” for histone modification, and “AND epigenetics^*^[tiab] NOT methylation[tiab] NOT histone^*^[tiab]” for other epigenetic biomarkers. In total, 1,444 articles were screened for these biomarkers in PubMed citations until December, 2019.

The following rules were applied to screen articles about CRC epigenetic biomarkers.

1. The article should contain clear statements like “Epigenetic modification (such as DNA methylation, histone modification, or other epigenetics modifications) is a biomarker/marker/indicator of CRC.” If the statement includes expressions like “can/may/has potential,” the corresponding data is included. This key statement can be searched in our database under “Description”.
2. Reviews or meta-analyses are excluded in the screening of CRC biomarkers.
3. If the article includes information about AUC/sensitivity/specificity or other assessment of the accuracy of the biomarker for prediction or classification of CRC, the value should be statistically significant.
4. Biomarkers from different articles have different IDs in our database, even if they share the same name, but with different clinical conditions for CRC, such as biomarker for diagnosis, prognosis, or treatment of CRC.
5. If both single and combinatorial biomarkers are included in one article, all the reported markers are given different IDs in our database.

**Figure 1:**
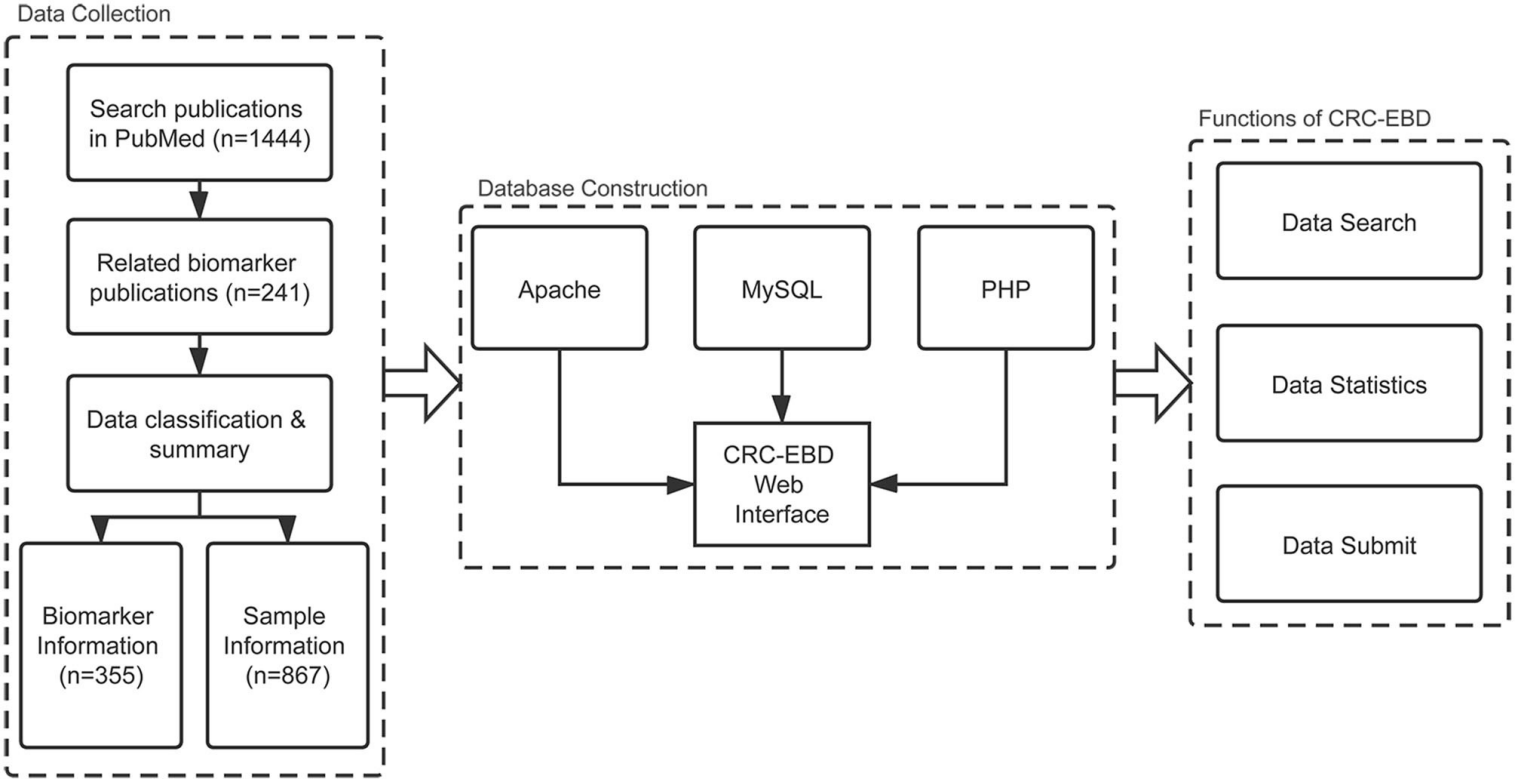
Pipeline of database construction.

We eventually selected 355 biomarkers, along with 694 records of sample information and 420 records of epigenetics information from the articles. The various cancer names in the original articles were uniformly changed to colorectal/colon/rectal cancer. A common format, as in “methylation of APC,” was adopted for all the biomarker names in CRC-EBD. All gene symbols and miRNA names were annotated as the official gene symbols from NCBI and miRBase. The biomarkers were also labeled with sample resources (blood, stool, and tissue) and clinical applications (diagnosis, prognosis, and treatment). Moreover, sample information of the patients (e.g., nationality, age, and TNM stage,) was collected for further analysis in personalized medicine. The pipeline of data collection, database construction, and functions of CRC-EBD is shown in Figure 1.

B/S (Browser/Server) structure and WAMP (Windows Server 2016 + Apache 2.4.39 + MySQL (10.4.6-MariaDB) + PHP 7.3.8) were used to construct the database. Users can access our database using their own browsers without installing other components. HTML and CSS were used to create the web pages and display the information. PHP and JavaScript were applied to connect the database and realize the search function. The data is stored in the MySQL database, which can be easily and quickly accessed. The charts in the statistics page were generated dynamically using ECharts (Li et al., 2018).

## Discussion

The epigenetic biomarkers in our online database can be searched by epigenetics name, epigenetics type, CRC subtype, biomarker type, and application. Epigenetics name searching mode allows users to enter the name of a gene, miRNA, or histone in a text box. Similarly, under CRC subtype searching mode, users can type in a text box the CRC subtypes or cancer names. Epigenetics types can be searched by DNA methylation, RNA methylation, histone modification, or others. Furthermore, users can select the biomarker type (diagnostic, prognostic, or therapeutic) and the application mode (blood, stool, tissue, or bowel lavage fluid) for their searches. The search result will be shown in a new webpage containing the list of biomarkers, and users can click each item for more detailed information.

**Figure 2:**
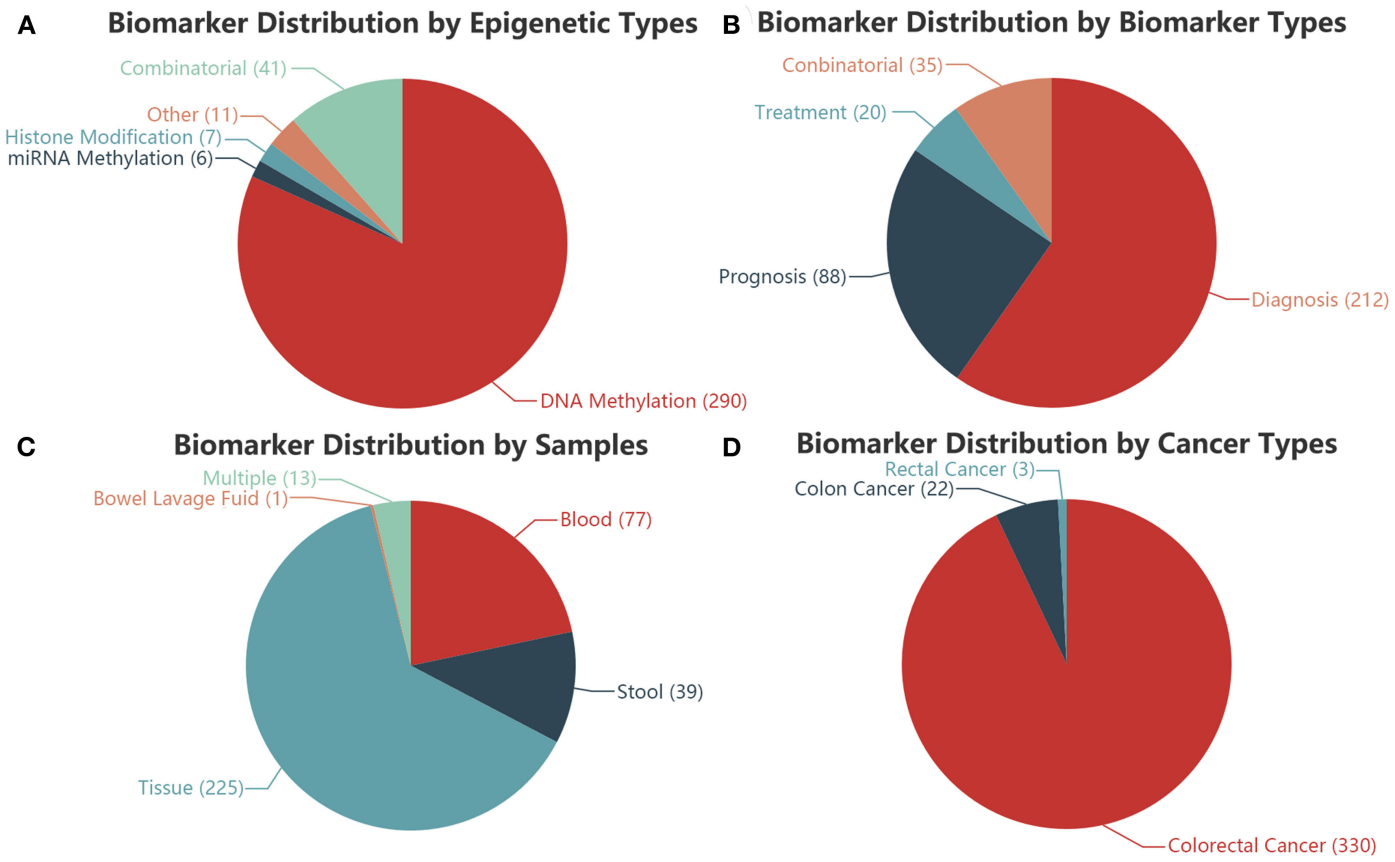
Distributions of the biomarkers in CRC-EBD. **(A)** Epigenetic types. **(B)** Biomarker types. **(C)** Sample types. **(D)** Cancer types.

Among the 355 epigenetics biomarkers in our CRC-EBD, 81.69% (290) of them are single DNA methylation biomarkers, whereas 11.52% are combinatorial (Figure 2A). Based on the clinical applications, 59.72% of the biomarkers are diagnostic, among which 9.86% are combinatorial for diagnosis, prognosis, or treatment (Figure 2B). 225 (63.38%) biomarkers are applied for tissue samples, 39 (10.99%) for stool, 77 (21.69%) for blood, and 13 (3.66%) for multiple sample types. A combined biomarker (miR-124-3, ZNF582-AS1, and SFRP1 methylation) is the only one reported for bowel lavage fluid detection (Figure 2C). 92.98% of the biomarkers in our database are applied for colorectal cancer research, demonstrating its prominence in the current field of studies (Figure 2D).

Six hundred and ninety four groups of samples in total are collected in the CRC-EBD: 457 are tissue samples (tumor samples and healthy samples), 73 are stool samples, 131 are serum/plasma samples or others. Though stool or blood samples are easier and more convenient to acquire, most of the previous studies are based on tissue samples directly connected to cancer genesis and progress.

CRC-EBD is the first online resource for epigenetic biomarkers of cancer. We will expand the database to other cancers in the future. This database will offer the users a systematic perspective on the heterogeneous cancer and promote epigenetics research on cancers.

## Data Availability Statement

Publicly available datasets were analyzed in this study. This data can be found at: http://www.sysbio.org.cn/EBD/.

## Author Contributions

XL, XZ, HZ, X-FS, and BS conducted and designed this study. XL, XZ, JC, BY, SR, and YL collected data and implemented the database. XL and SR wrote the manuscript. BS supervised the project. All authors reviewed and approved the paper for publication.

## Conflict of Interest

The authors declare that the research was conducted in the absence of any commercial or financial relationships that could be construed as a potential conflict of interest.
